# Predicting drug–Protein interaction with deep learning framework for molecular graphs and sequences: Potential candidates against SAR-CoV-2

**DOI:** 10.1371/journal.pone.0299696

**Published:** 2024-05-10

**Authors:** Weian Du, Liang Zhao, Rong Wu, Boning Huang, Si Liu, Yufeng Liu, Huaiqiu Huang, Ge Shi

**Affiliations:** 1 Department of Dermatology, Third Affiliated Hospital of Sun Yat-sen University, Guangzhou, China; 2 Shenzhen Health Development Research and Data Management Center, Shenzhen, China; 3 School of Finance, Shanghai University of Finance and Economics, Shanghai, China; 4 Department of Cosmetic and Plastic Surgery, The Sixth Affiliated Hospital, Sun Yat-sen University, Guangzhou, China; Concordia University, CANADA

## Abstract

The severe acute respiratory syndrome coronavirus 2 (SARS-CoV-2) caused the COVID-19 disease, which represents a new life-threatening disaster. Regarding viral infection, many therapeutics have been investigated to alleviate the epidemiology such as vaccines and receptor decoys. However, the continuous mutating coronavirus, especially the variants of Delta and Omicron, are tended to invalidate the therapeutic biological product. Thus, it is necessary to develop molecular entities as broad-spectrum antiviral drugs. Coronavirus replication is controlled by the viral 3-chymotrypsin-like cysteine protease (3CLpro) enzyme, which is required for the virus’s life cycle. In the cases of severe acute respiratory syndrome coronavirus (SARS-CoV) and middle east respiratory syndrome coronavirus (MERS-CoV), 3CLpro has been shown to be a promising therapeutic development target. Here we proposed an attention-based deep learning framework for molecular graphs and sequences, training from the BindingDB 3CLpro dataset (114,555 compounds). After construction of such model, we conducted large-scale screening the *in vivo/vitro* dataset (276,003 compounds) from Zinc Database and visualize the candidate compounds with attention score. geometric-based affinity prediction was employed for validation. Finally, we established a 3CLpro-specific deep learning framework, namely GraphDPI-3CL (AUROC: 0.958) achieved superior performance beyond the existing state of the art model and discovered 10 molecules with a high binding affinity of 3CLpro and superior binding mode.

## 1. Introduction

The outbreak of the coronavirus that causes severe acute respiratory syndrome in 2019 has resulted in a global pandemic of severe pneumonia-like disease and known as COVID-19. During the infection process, the coronavirus genome utilized the host ribosomes and translated into a large polypeptide (PP) chain, which is then cleaved by two proteases, papain like proteases (PLpro) and 3-chyomotrypsin like protease (3CLpro), to generate several non-structural proteins (NSPs) required for viral replication [1]. PLpro and 3CLpro (M^pro^) cleave the PP chain into 16 NSPs, and 11 NSPs are generated by the 3CLpro [2,3]. M^pro^ mediate viral replication by cleaving viral polyprotein precursors at specific sites [4] and inhibition of M^pro^ can block the synthesis of viral proteins, suggesting that 3CLpro can be one of the target candidates for developing anti-COVID-19 drugs [5,6].The 3CLpro protein sequences for SARS-CoV-2 are publicly available on the Protein Data Bank and BindingDB 3CLpro dataset [4]. Herein, we have selected 3CLpro of SARS-CoV-2 as a target candidate to identify potential inhibitors since 3CLpro is indispensable for viral replication. Furthermore, numerous isoflavone glycosides were isolated from soybean seed hypocotyls, demonstrating the capacity to prevent coronavirus-induced cytopathological effects [7].

Issahaku et al. elucidate the binding mechanism of MRTX1133, a potential KRASG12D inhibitor, offering insights that could inform the targeting of analogous protein interaction domains within SARS-CoV-2 [8]. Similarly, the exploration of dual COX and LOX inhibition by compounds in Indian spices suggests an innovative therapeutic approach to viral mitigation by modulating the inflammatory response, which may be applicable to SARS-CoV-2 [9]. Furthermore, the molecular interactions of bioactive compounds as potential SARS-CoV-2 protease inhibitors highlight a promising direction for multi-targeted antiviral strategies [10]. This is complemented by research into arylhydrazone derivatives, indicating a new frontier for antiviral drug development relevant to SARS-CoV-2 [11]. Additionally, the molecular structure elucidation of bioactive compounds provides a foundational framework for structural-based drug discovery efforts aimed at combating SARS-CoV-2 [12]. Finally, a comprehensive review underscores the potential of nutraceuticals in enhancing immunity against respiratory viruses, including SARS-CoV-2 [13].

Recently, there has been a surge of AI in drug discovery over the past decade, which is still gaining popularity. AI has the potential to revolutionize medication research, such as the proteome of Alphafold DB [14,15]. Through technical advancements such as the use of neural networks to design compounds and the application of knowledge graphs to comprehend target biology, AI-enabled drug development [16,17] has progressed significantly over the last few years. Several AI-native drug discovery businesses have advanced compounds into clinical trials, indicating considerably quicker schedules and lower costs in certain cases, setting high expectations in the R&D sector [18,19]. In addition, several well-known pharmaceutical corporations have created discovery agreements with AI firms in order to investigate the technology. Despite this advancement, AI in drug discovery is still in its infancy, with many unanswered issues concerning its effect and future possibilities.

In our research, we initially introduced an innovative deep learning framework based on an attention mechanism, specifically designed for molecular graphs and sequences, which was trained using the BindingDB 3CLpro dataset, consisting of 114,555 compounds. Following the development of this model, we performed extensive screening on a combined in vivo and in vitro dataset comprising 276,003 compounds from the Zinc Database, and we subsequently visualized the potential candidate compounds, highlighting them with their respective attention scores. To validate our findings, we applied a geometric-based approach for predicting binding affinity. Culminating our efforts, we established a specialized deep learning framework tailored for 3CLpro, denoted as GraphDPI-3CL, which yielded an impressive AUROC of 0.958. This framework enabled us to identify 10 molecules that demonstrated high binding affinity toward 3CLpro, along with an exceptional binding mode.

## 2. Materials and methods

### 2.1 Data preprocessing

The Lipinski parameters for molecules were used for druggability evaluation, which were calculated using RDKit [20]. The molecules in Zinc database were filtered by Lipinski rules including: 1) Molecular weight is less than 500 Da; 2) Number of H bond donors (including hydroxyl, amino, etc.) does not exceed 5; 3) Number of H bond acceptors in the compound does not exceed 10; 4) logarithm of the fat-water partition coefficient (logP) is between -2 and 5; 5) Number of rotatable bonds in the compound does not exceed 10. Detecting problematic candidates at early stage can significantly reduce wasted time and resources, and thus the ADME-related properties were calculated and filtered by using the QikProp [21] program in normal mode. The overall 46 related descriptors were chosen to filter our natural products. Some important descriptors include: 1) the CNS parameter: predicted central nervous system activity on a −2 (inactive) to +2 (active) scale. 2) QPlogS: the logarithm of aqueous solubility (range for 95% of drugs: -6.0 to 0.5); logS is the concentration of the solute in a saturated solution that is in equilibrium with the crystalline solid. 3) QPlogKHSA: the logarithm of predicted binding constant to human serum albumin (range for 95% of drugs: -1.5 to 1.2). 4) Human Oral Absorption: predicted qualitative human oral absorption: 1, 2, or 3 indicating for low, medium, or high; the assessment uses a knowledge-based set of rules, including checking for suitable values of Percent Human Oral Absorption, number of metabolites, number of rotatable bonds, logP, solubility, and cell permeability [22].

### 2.2 Data encoding

The graph representation and descriptor of the filtered molecules was generated by using RDKit [20]. The molecule with *N*_*a*_ atoms can be represented by a graph *G* = {*V*, *E*}, where the node *v*_*i*_∈*V*, *i* = 1, 2, …, *N*_*a*_, is corresponded to the *i*-th non-hydrogen atom in the molecule candidates, and each edge *e*_*i*,*i*_∈*E*, *i*,*j* ∈{1, 2, …, *N*_*a*_}, is corresponded to a chemical bond between the *i*-th and the *j*-th atoms [23]. The descriptor [24] of each atom was represented as a 34-dimension vector, indicating atom type, degree of atom, formal charge, number of radical electrons, hybridization type, aromaticity, number of hydrogen linking atoms, chirality and configuration in molecular feature.

### 2.3 Model architecture of GraphDPI-3CL

The proposed model is consisting of two major parts, message-passing neural network (MPNN) module [25] and self-attentive bidirectional long short-term memory (BiLSTM_Atten) [26]. The MONN is designed for the semi-supervised node classification problem such as molecular graph representation. The molecule was denoted as graph representation *G* = {*V*, *E*}, each node *V* represented as a n-dimensional feature vector and each edge *E* is the set of covalent bonds as an adjacency matrix *E* ∈ *R*^*m × m*^. The MONN module aggregates all atoms and bonds information at molecule level with the following equation.

$$H^{\left(l+1\right)}=f(H^{\left(l\right)},A)=\sigma ({\tilde{D}}^{-\frac{1}{2}}\tilde{A}{\tilde{D}}^{-\frac{1}{2}}H^{\left(l\right)}W_{3}^{\left(l\right)})$$

where *H*^*(l)*^ ∈ *R*^*n × m*^, is the output the *l*-th hidden layer, *W*^*(l)*^ ∈ *R*^*n × n*^ is the weight matrix for the *l*-th neural network layer, *D*∈ *R*^*m × m*^ is diagonal matrix and σ () is rectified linear unit (ReLU) activation function, $\tilde{A}=A+I,I$ is the identity matrix. In our proposed model, n = 34 and the final output of MONN, *O* ∈ *R*^*n × s*^, where *s* is the length of the molecule sequence. After processing of MONN, the output was fed into the module of BiLSTM_Atten with following equation.

$$\begin{array}{l} \vec{h_{i}}=LSTM(t_{i},\vec{h_{i-1}})\\ \overset{\leftarrow }{h_{i}}=\overset{\leftarrow }{LSTM}(t_{i},\overset{\leftarrow }{h_{i+1}})\\ h_{i}=(\vec{h_{i}},\overset{\leftarrow }{h_{i}})\\ H=(h_{0},h_{i}\cdots h_{n}) \end{array}$$

where $\vec{h_{i}}$ is the forward-directed hidden layer output and $\overset{\leftarrow }{h_{i}}$ is the backward-directed hidden layer output, thus $h_{i}$ is able to collect the bidirectional information dependency between adjacent tokens in a molecule. To visualize the atom contribution for the interaction prediction, we introduce multi-head self-attention mechanism into BiLSTM. The attention block takes the whole LSTM hidden layer *H* output as input following the equation as

$$A^{m}=softmax(\mathrm{MLP}\left(H^{T}\right))$$

where hidden layer number of MLP is 512 in this study and we obtained the weighted sums by multiplying the annotation matrix *A*^*m*^ and LSTM hidden states *H*. After the molecule feature extraction, we fed the concatenation of last hidden layer of *H* and the sums of attention score into 2 fully connected layer for classification and employed cross entropy loss function and Adam optimizer for the training.

$$L_{CE}(\Theta )=-\sum_{i=1}^{N}\;(y_{i}log(\sigma (o_{i}))+(1-y_{i})log(1-\sigma (o_{i})))+\frac{\lambda }{2}∥\Theta {∥}_{2}^{2}$$

where Θ is the set of all weight matrices and bias vectors, *N* is the number of molecules in training dataset, λ is L2 regularization for training stride punishment.

**Fig 1:**
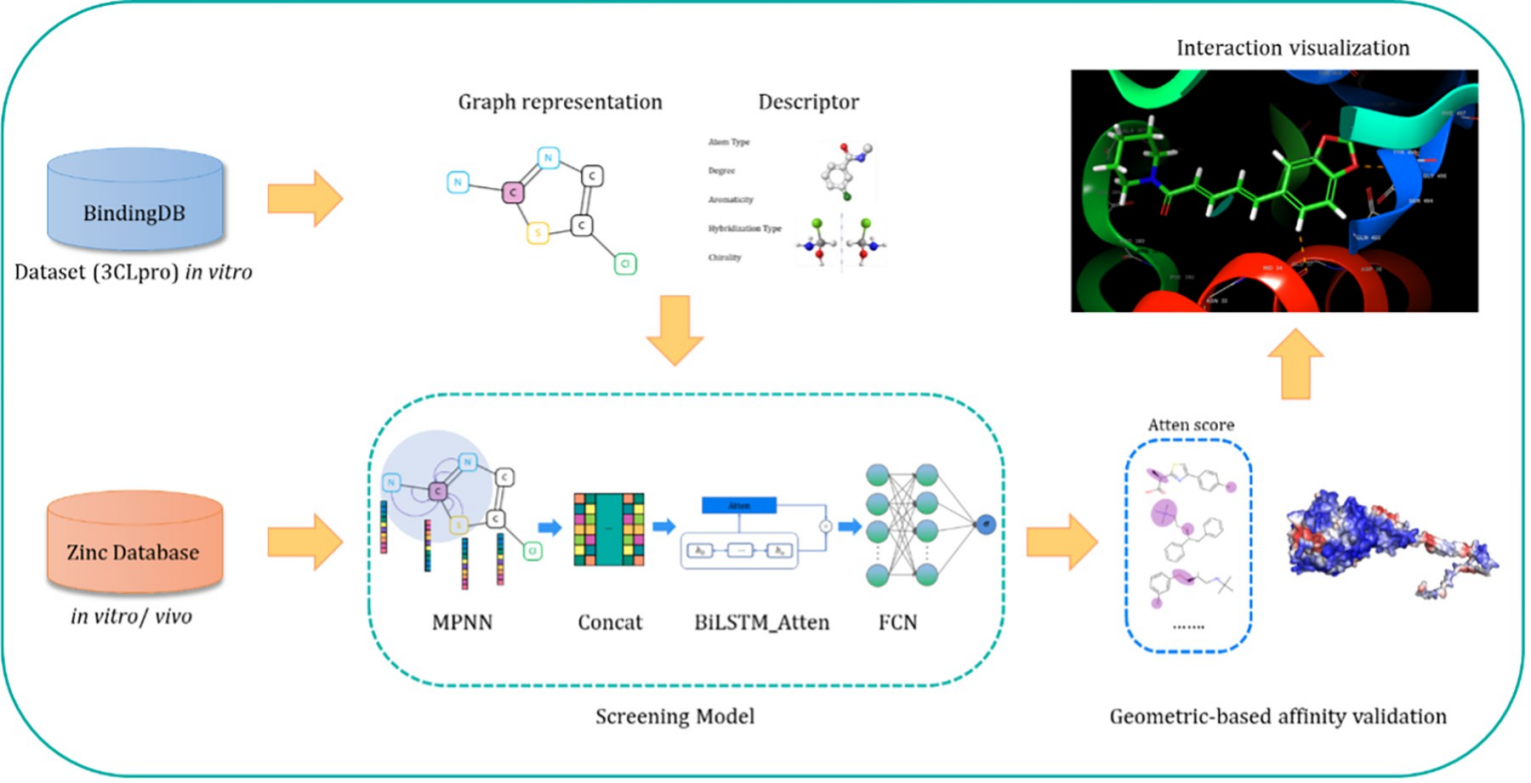
The framework of machine learning screening and geometric-based affinity prediction.

GraphDPI-3CL is composed of three encoder layers and three decoder layers (Fig 1), which are instrumental in capturing the complex relationships within the protein-drug interaction data. Each atom is represented in a 64-dimensional vector space, allowing the model to effectively learn the nuances of molecular structures. The model utilizes a multi-head attention mechanism with eight attention heads, facilitating the simultaneous processing of different representation subspaces and enhancing the model’s ability to focus on various parts of the input graph. For the training process, we have chosen a learning rate of 1e-4 and a weight decay of 1e-4 to ensure stable convergence while preventing overfitting. The batch size is set to 8, balancing the computational efficiency with the ability to generalize across the dataset. To further aid in the model’s generalization, we have implemented a dropout rate of 0.2, which helps mitigate the risk of co-adaptation of neurons.

### 2.4 Model comparison

The baseline model has been proposed for performance comparison including deep learning-based model such as DrugVQA [27], TransformerCPI [28] and machine learning-based model such as XGBoost [29], Gradient Boosted Trees. For fair comparison, we adopted simplified version of DrugVQA without protein structure information (DrugVQA-seq) provided by author. TransformerCPI was implemented and pretrained by configuration of author. The machine learning-based methods including XGBoost, Gradient Boosted Trees was performed by RDKit and Knime platform. We employed 5-fold cross-validation strategy for the evaluation of performance. The predictive performance of the model is assessed with the area under receiver operating characteristic (AUROC), Accuracy (Acc), Specificity (Spec), Recall and Matthews correlation coefficient (MCC).


$$\begin{array}{c} ACC=\frac{TP+TN}{TP+FP+TN+FN}\\ SN=\frac{TP}{TP+FN}\\ SP=\frac{TN}{TN+FP}\\ MCC=\frac{TP\times TN-FP\times FN}{\sqrt{\left(TN+FN)(TN+FP)(TP+FN)(TP+FP\right)}} \end{array}$$

### 2.5 Interaction visualization

MaSIF [30] is a deep learning framework to capture fingerprints that are important for geometric biomolecular interactions, which is a compound-protein interaction prediction software with high precision. After the large-scale screening, the premilarily positive molecule will be input to MaSIF to visualize the compound-protein interaction and interaction analysis and the MaSIF tool can be assessed at https://github.com/LPDI-EPFL/masif.

## 3. Results

### 3.1. Machine learning model

The efficacy of the proposed computational models is delineated in Table 1. Among the models evaluated, GraphDPI-3CL exhibited a superior performance, as evidenced by its attainment of the highest values in key metrics, including the area under the receiver operating characteristic curve (AUROC), specificity (Spec), recall, and Matthew’s correlation coefficient (MCC). In comparison, TransformerCPI was notable for achieving the highest accuracy.

**Table 1:** The performance of model.

Model	AUROC	Acc	Sen	Spec	Recall	MCC
GraphDPI-3CL	**0.958**	0.938	**0.957**	**0.912**	**0.957**	**0.870**
DrugVQA-seq	0.910	0.888	0.885	0.875	0.863	0.815
TransformerCPI	0.912	**0.951**	0.901	0.908	0.919	0.868
XGBoost	0.726	0.729	0.711	0.699	0.710	0.602
Gradient Boosted Trees	0.701	0.705	0.689	0.680	0.701	0.612

Area under receiver operating characteristic (AUROC), Accuracy (Acc), Sensitivity (Sen), Specificity (Spec), Recall and Matthews correlation coefficient (MCC).

**Fig 2:**
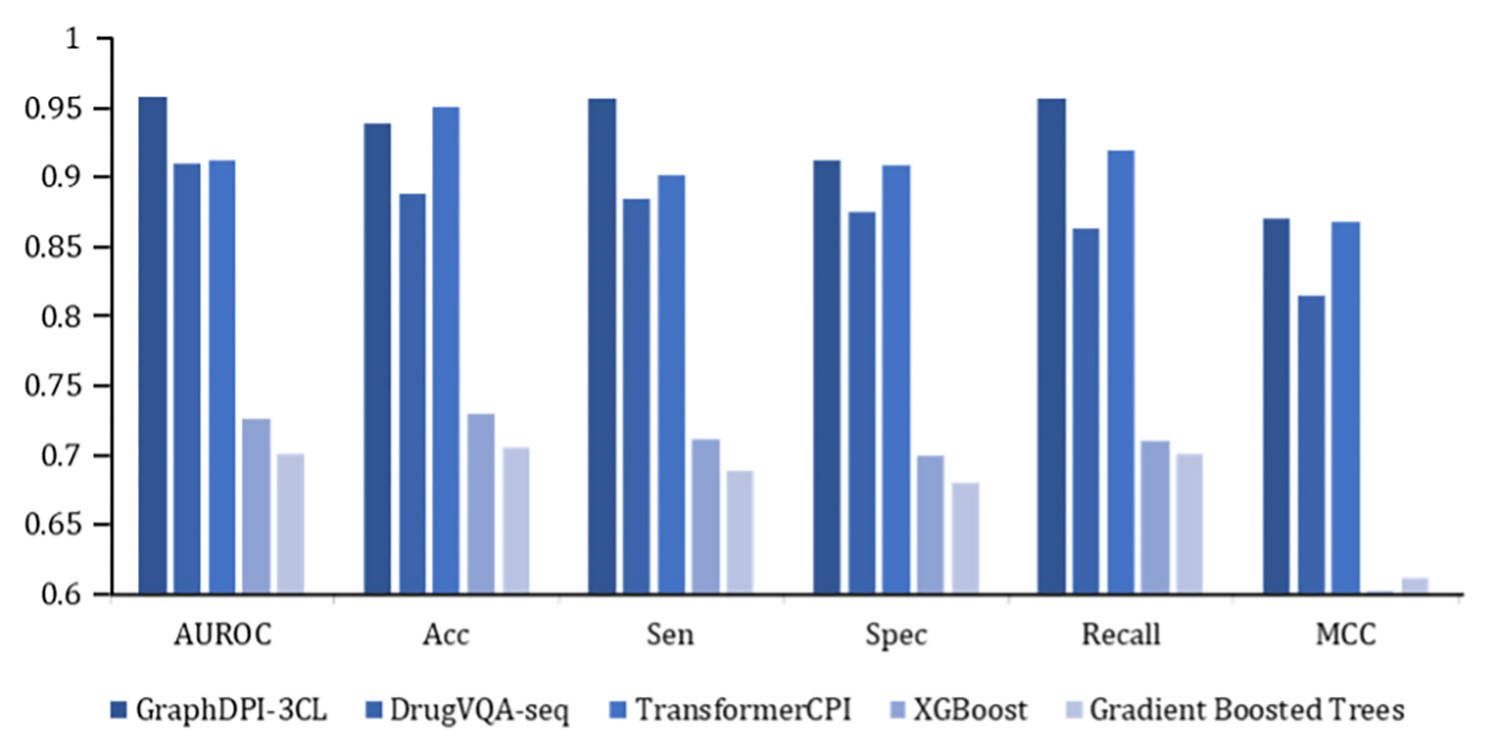
The performance metric of the proposed 3CLpro-specific affinity prediction model.

GraphDPI-3CL outperformed both deep learning-based models (Fig 2), such as DrugVQA and TransformerCPI, and machine learning-based models, such as XGBoost and Gradient Boosted Trees, across several evaluation metrics. This superior performance of GraphDPI-3CL is particularly evident in its predictive precision and ability to correctly classify both positive and negative instances, as reflected by its high specificity and recall. Leveraging the strengths of GraphDPI-3CL, we conducted large-scale virtual screening using the Zinc Database, incorporating a druggability assessment to refine our search. This process culminated in the identification of the top 15 candidate molecules. Subsequent geometric-based affinity validation and interaction analysis were performed on these candidates, leading to the discovery of four molecules characterized by a high binding affinity for 3CLpro and an optimal binding mode. These results underscore the potential of GraphDPI-3CL as a robust tool in the identification of promising therapeutic compounds. The integration of GraphDPI-3CL into the drug discovery pipeline could significantly enhance the efficiency and accuracy of virtual screening processes.

### 3.2. Compound-protein interaction visualization

The reliability of our docking methodology was established through a re-docking assessment using the co-crystallized ligands of Mpro. Employing Glide software (v2019.2), we compared the re-docked conformations with their respective co-crystallized counterparts. The root-mean-square deviation (RMSD) values obtained from this comparison were 2.5 Å and 2.8 Å, providing evidence of the docking protocol’s accuracy and reliability [31]. Further validation of our computational approach is evident in the docking results detailed in Table 2. Out of the screened molecular candidates, 15 exhibited strong binding to Mpro, demonstrating notable binding energies. Impressively, 10 of these candidates surpassed the binding affinity of the co-crystallized ligand, N-acetylglucosamine (NAG), suggesting that these molecules possess the potential to act as biologically active ligands against Mpro. This is a significant finding, as it not only validates our docking procedures but also identifies promising candidates for further experimental investigation.

**Table 2:** Docking results of top 15 molecule candidates toward M^pro^.

Num	MaSIF-Ligand score	Glide ligand efficiency	Glide energy (kcal mol^−1^)
1	-7.12	-0.264	-53.096
2	-3.779	-0.18	-41.554
3	-1.634	-0.082	-34.937
4	-6.549	-0.243	-49.342
5	-3.831	-0.167	-33.857
6	-7.079	-0.244	-50.767
7	-5.346	-0.206	-41.892
8	-6.642	-0.289	-46.951
9	-5.692	-0.271	-38.711
10	-5.048	-0.158	-29.765
11	-5.735	-0.239	-39.926
12	-5.092	-0.145	-48.435
13	-4.17	-0.144	-37.308
14	-5.193	-0.192	-36.767
15	-3.466	-0.144	-26.592
NAG	-5.471	-0.244	-36.592

**Fig 3:**
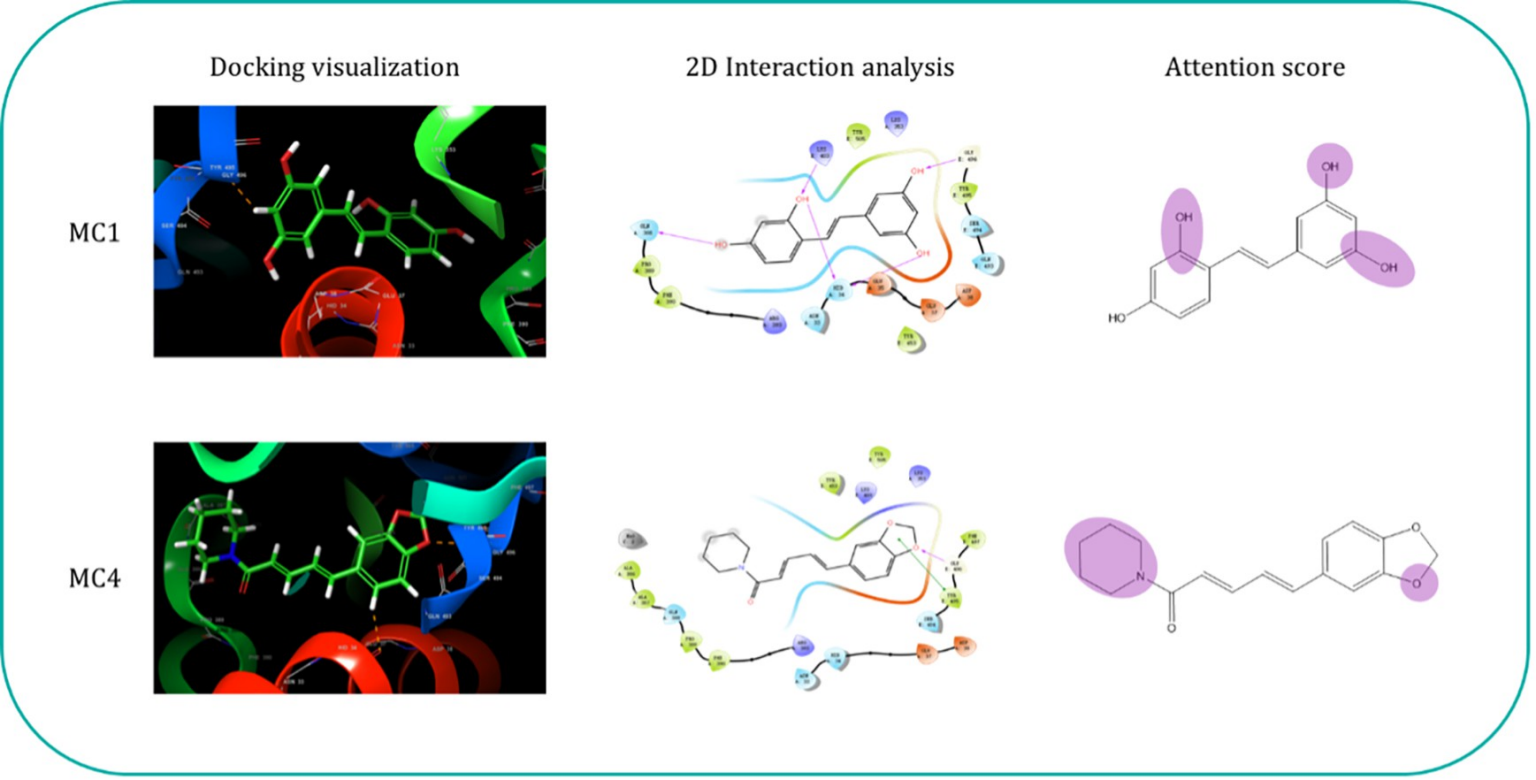
Model interpretation analysis reveals GraphDPI-3CL can capture binding affinity weight on ligand and providing insightful guidance for downstream quantitative structure-activity relationship analysis.

The screening process revealed that the molecular candidates bound to 3CLpro exhibited free binding energy values spanning from -53.096 to -25.819 kcal/mol, which were benchmarked against the co-crystallized ligand NAG, with a known binding energy of -36.592 kcal/mol. Notably, molecules 1 and 4, as depicted in Fig 3, demonstrated particularly favorable binding energies of -52.096 kcal/mol and -49.342 kcal/mol respectively. These values suggest a higher binding affinity to 3CLpro, potentially indicating stronger inhibitory action on the enzyme compared to NAG. The precise binding interactions of these candidates were further elucidated using Glide software’s 2D interaction analysis. This analysis highlighted two critical hydrogen bonds formed with the GLY496 residue, which may significantly contribute to the observed high binding affinity for 3CLpro of SARS-CoV-2. The importance of these interactions is supported by the attention weights from the self-attention layer of our computational model, which showed alignment with the Glide 2D interaction findings. This consistency underscores the model’s ability to capture essential mutual information between the molecule candidates and the target protein, which could offer valuable insights and direction for the optimization of lead compounds.

## 4. Conclusion

The ongoing COVID-19 pandemic continues to exert a profound global impact, underscoring the urgent need for effective vaccines and therapeutic agents. This urgency has galvanized efforts to mine the vast chemical space for safe and efficacious treatments. In our research, we have introduced an innovative attention-based deep learning framework tailored for the analysis of molecular graphs and sequences. Trained on a comprehensive BindingDB dataset specific to 3CLpro that includes 114,555 compounds, our model is designed to effectively discern high-affinity drug candidates. Upon establishing this model, we embarked on a large-scale screening of an extensive in vivo/in vitro dataset, comprising 276,003 compounds from the Zinc Database. To ensure a focused approach, we applied attention mechanisms to rank and visualize the candidate compounds based on their predicted binding affinity, highlighted by their respective attention scores. To validate the predictive outcomes, we employed geometric-based affinity prediction techniques. The culmination of our efforts is the creation of a deep learning framework specialized for 3CLpro, which we have named GraphDPI-3CL. This model boasts a remarkable AUROC score of 0.958, underscoring its predictive prowess. Despite the promising performance of GraphDPI-3CL, the framework is not without its limitations. One significant drawback is its potential to miss potential inhibitors that could be detected through methods that consider the three-dimensional structure of proteins. The model’s current design does not explicitly incorporate chemical bond information, which could lead to less detailed molecular representations. The absence of direct modeling of chemical bond types and configurations within the molecules means that the framework might overlook subtle but critical aspects of molecular interactions and activity, which are essential for a comprehensive understanding of the intricacies involved in molecular binding processes. Moreover, to effectively adapt our model to new protein targets, it is essential to compile a comprehensive dataset of protein-ligand interactions specific to each target, which will be used to retrain the model, leveraging its existing architecture. This retraining enables the model to learn the unique interaction patterns of the new protein. Following this, it is crucial to validate and test the model’s predictions against known data and, where possible, through experimental assays to ensure its predictive accuracy and reliability for the new target.

Our framework has facilitated the identification of 10 molecular candidates that not only possess high binding affinity to 3CLpro but also exhibit optimal binding modes, which are critical for effective inhibition of the viral enzyme. While several universal deep learning-based models have been developed for drug-protein interaction prediction, GraphDPI-3CL sets itself apart by being a bespoke model for 3CLpro, outperforming existing state-of-the-art models in this domain. Consequently, GraphDPI-3CL represents a powerful tool for large-scale screening of molecule candidates targeting 3CLpro, with the potential to expedite the drug discovery journey in the fight against SARS-CoV-2.
